# Neuroprotective Actions of Methylene Blue and Its Derivatives

**DOI:** 10.1371/journal.pone.0048279

**Published:** 2012-10-31

**Authors:** Ethan Poteet, Ali Winters, Liang-Jun Yan, Kyle Shufelt, Kayla N. Green, James W. Simpkins, Yi Wen, Shao-Hua Yang

**Affiliations:** 1 Department of Pharmacology and Neuroscience, Institute for Alzheimer’s Disease and Aging Research, University of North Texas Health Science Center at Fort Worth, Fort Worth, Texas, United States of America; 2 Department of Chemistry, Texas Christian University, Fort Worth, Texas, United States of America; Universidade Federal do Rio de Janeiro, Brazil

## Abstract

Methylene blue (MB), the first lead chemical structure of phenothiazine and other derivatives, is commonly used in diagnostic procedures and as a treatment for methemoglobinemia. We have previously demonstrated that MB could function as an alternative mitochondrial electron transfer carrier, enhance cellular oxygen consumption, and provide protection *in vitro* and in rodent models of Parkinson’s disease and stroke. In the present study, we investigated the structure-activity relationships of MB *in vitro* using MB and six structurally related compounds. MB reduces mitochondrial superoxide production via alternative electron transfer that bypasses mitochondrial complexes I-III. MB mitigates reactive free radical production and provides neuroprotection in HT-22 cells against glutamate, IAA and rotenone toxicity. Distinctly, MB provides no protection against direct oxidative stress induced by glucose oxidase. Substitution of a side chain at MB’s 10-nitrogen rendered a 1000-fold reduction of the protective potency against glutamate neurototoxicity. Compounds without side chains at positions 3 and 7, chlorophenothiazine and phenothiazine, have distinct redox potentials compared to MB and are incapable of enhancing mitochondrial electron transfer, while obtaining direct antioxidant actions against glutamate, IAA, and rotenone insults. Chlorophenothiazine exhibited direct antioxidant actions in mitochondria lysate assay compared to MB, which required reduction by NADH and mitochondria. MB increased complex IV expression and activity, while 2-chlorphenothiazine had no effect. Our study indicated that MB could attenuate superoxide production by functioning as an alternative mitochondrial electron transfer carrier and as a regenerable anti-oxidant in mitochondria.

## Introduction

Neurological disorders are estimated to affect as many as 1 billion people globally [1]. The cost of dementia alone is estimated at $100 billion annually in the United States [1]. Increased oxidative stress has been recognized as a common theme of many neurological disorders including Alzheimer’s disease, Parkinson’s disease, and stroke [2], [3], [4]. Antioxidative strategies have been extensively explored for the treatment of various neurological disorders with many of the compounds demonstrating neuroprotection in multiple *in vitro* and *in vivo* models. However, none of the identified antioxidants have proven to be effective for the treatment of any neurodegenerative disease in clinical settings [5], [6], [7]. It is therefore important and practical to examine alternative strategies for reducing oxidative stress besides traditional antioxidants.

Methylene blue (MB), the very first lead chemical structure of phenothiazine and other derivatives, has been used for diagnostic procedures and the treatment of multiple disorders; including methemoglobinemia, malaria, and cyanide and carbon monoxide poisoning [8], [9]. Recently, we have shown MB to be neuroprotective in a variety of mitochondria targeted cytotoxicity paradigms [10]. MB retains its protective activity in *in vivo* models of stroke, Parkinson’s disease, and optic neuropathy [10], [11]. Importantly, MB is distinct from traditional antioxidants in that it provides no protection against direct oxidative insult of H_2_O_2_ produced by glucose oxidase [10].

**Figure 1:**
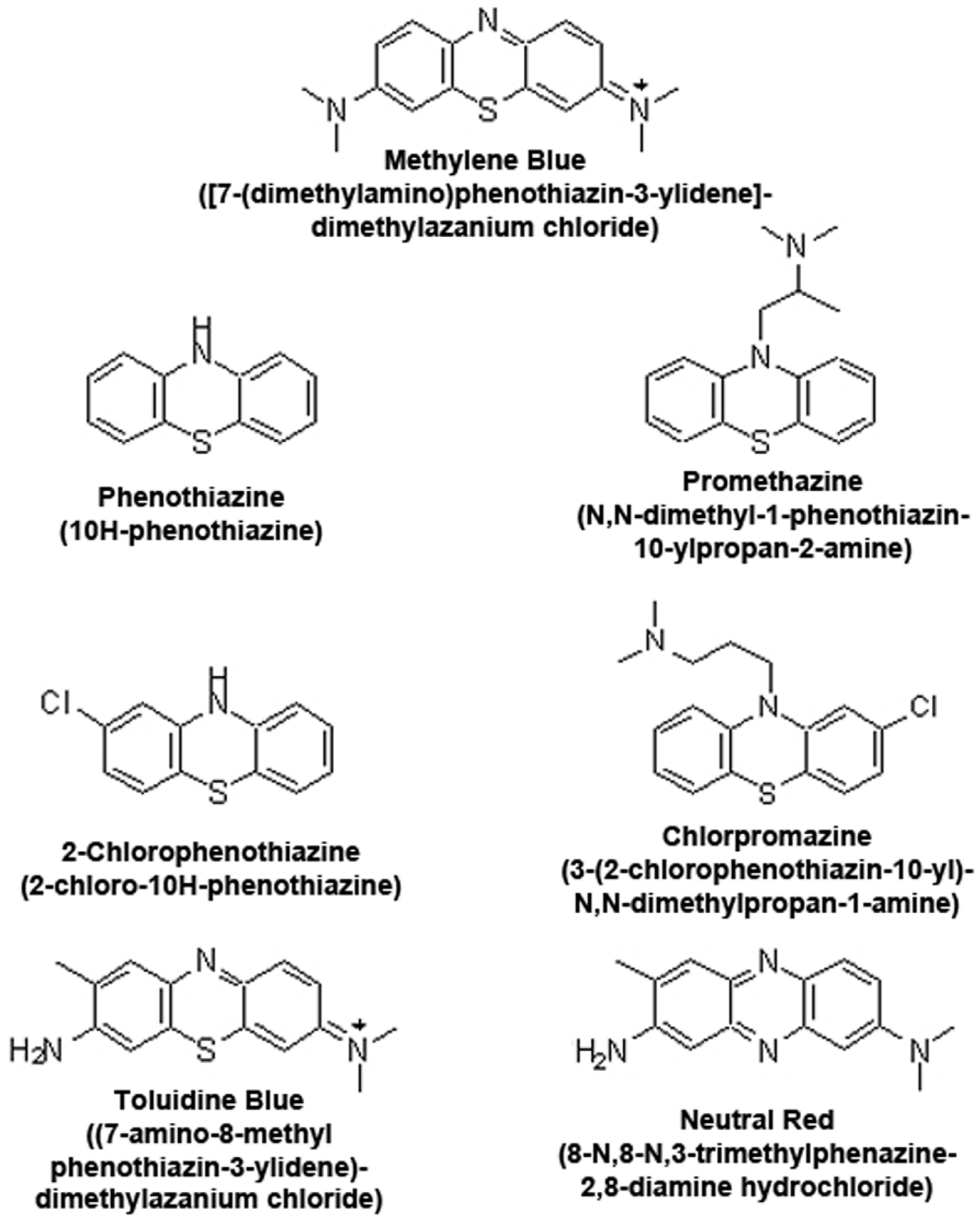
Chemical Structure and IUPAC designation of MB and its related compounds.

MB has long been known as an electron carrier, which is best represented by MB’s action to increase the rate of cytochrome c reduction in isolated mitochondria [12]. Through this shunt, MB causes an increase in cellular oxygen consumption and a corresponding decrease in anaerobic glycolysis *in vitro* and *in vivo* [10], [13], [14]. In addition, chronic exposure to MB results in increased activity and expression of mitochondria complex IV [15], [16]. In this study, we determined the structure-activity relationship of MB using MB and six other derivatives: toluidine blue O (TB), 2-chlorophenothiazine, phenothiazine, promethazine, chlorpromazine, and neutral red (NR). These derivatives comprise three major modifications of MB (Figure 1): 1) side chain deletions at positions 3 and 7 (phenothiazine and 2-chlorophenothiazine), 2) substitution of a side chain at position 10 (chlorpromazine and promethazine), 3) substitution of sulfur at position 5 with nitrogen (neutral red). Our study demonstrated that MB has a distinct action as an alternative mitochondrial electron transfer carrier and a re-generable anti-oxidant in the mitochondria and hence may provide neuroprotective effects for various neurological disorders.

## Materials and Methods

### Cell Culture

HT-22 cells, a murine hippocampal cell line derived from the mouse hippocampal HT-4 cell line, were the generous gift of Dr. David Schubert (Salk Institute, San Diego, CA) [17]. Cells were maintained in high glucose DMEM (HyClone, Logan, UT) with 1 mM pyruvate and 4 mM glutamine media supplemented with 10% FBS (Equitech Bio, Lewisville, TX), and Pen/Strep in monolayers in 100 mm Greiner tissue culture dishes (Greiner, Orlando, FL) at standard cell culture conditions (5% CO_2_, 95% air). Medium were changed three times weekly and back cultured at confluence (every 3–5 days). Cells were observed with a phase-contrast microscope (Zeiss Invertoskop 40°C). HT-22 cells were used between passages 10–30.

**Figure 2:**
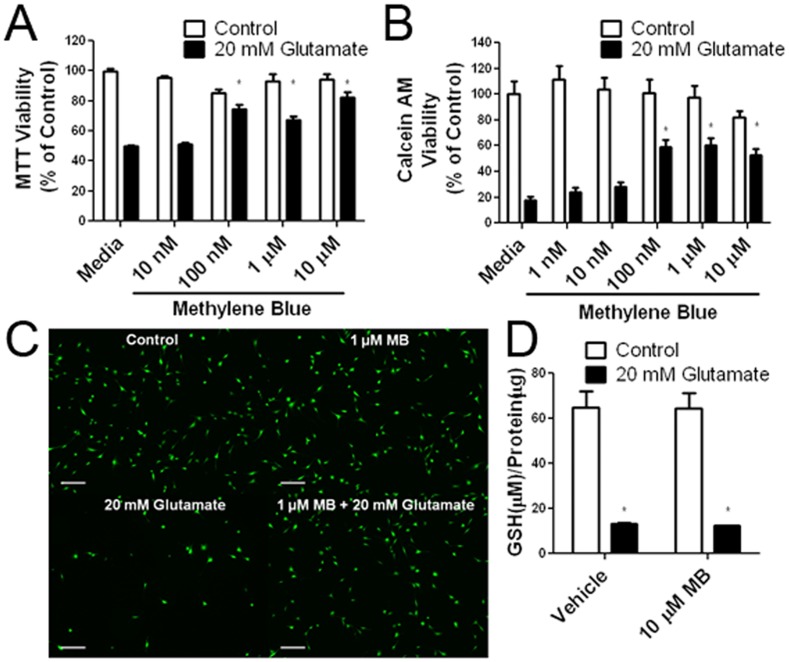
Neuroprotective effect of MB against glutamate neurotoxicity in HT-22 cells. (A) MTT viability assay (B) and Calcein AM viability assay at 12 hours after 20 mM glutamate insult demonstrate the dose dependent neuroprotective effect of MB. * p<0.05 compared to 20 mM glutamate media (C) Representative Calcein AM fluorescent images depict the neuroprotective effect of MB (1 µM) in HT-22 cells after 12 hours exposure of 20 mM glutamate (scale bar 200 µm). (D) Total GSH assay demonstrates that MB has no effect on the glutamate-induced GSH depletion. * p<0.05 compared to vehicle control.

**Table 1:** EC50 values for viability, ROS, and membrane potential of the MB related compounds in the HT-22 glutamate toxicity assay.

Drug	Viability (nM)	ROS(nM)	Membrane Potential (nM)
Toluidine Blue	1.75	0.11	1.54
2-Chlorophenothiazine	4.57	84.14	45.09
Methylene Blue	17.81	20.37	17.72
Phenothiazine	18.99	57.21	21.18
Promethazine	1067.00	9687.00	213.70
Chlorpromazine	2192.00	2148.00	3270.00
Neutral Red	5836.00	15760.00	8958.00

### Cell Viability Assay

Cell viability was determined by Calcein AM and MTT assays. For the Calcein AM assay, HT-22 cells were seeded at a density of 3,000 cells/well and were incubated overnight in 96-well plates in 100 µl of DMEM (high glucose with 1 mM pyruvate and 10% FBS). Varying concentrations of MB or its derivatives and 20 mM glutamate were added to each well and incubated for 12 hours at 37°C with 5% CO_2_. After 12 hours, media was removed and replaced with a 1 µM solution of Calcein AM in PBS. Cells were incubated for 5 minutes at 37°C and fluorescence was measured using a Tecan Infinite F200 plate reader (excitation 485 emission 530). For the MTT assay, HT-22 cells were seeded into 96-well, flat-bottomed plates at a density of 3000 cells/well in 100 µl DMEM (high glucose, 1 mM pyruvate, 10% FBS) and allowed to attach overnight. Varying concentrations of drug and 20 mM glutamate (or media for control wells) was then added to each well. Plates were incubated for 12 hours at 37°C with 5% CO_2_. Plates were removed from the incubator and 20 µl MTT (5 mg/ml in PBS) was added per well. The plates were agitated gently to mix the MTT into the media and then returned to the incubator for 2 hours. After 2 hours the media was removed and 100 µl of DMSO was added to each well. The plate was mixed by gentle agitation and the absorbance was measured (560 nm with a reference of 670 nm) with a Tecan Infinite F200 plate reader.

**Figure 3:**
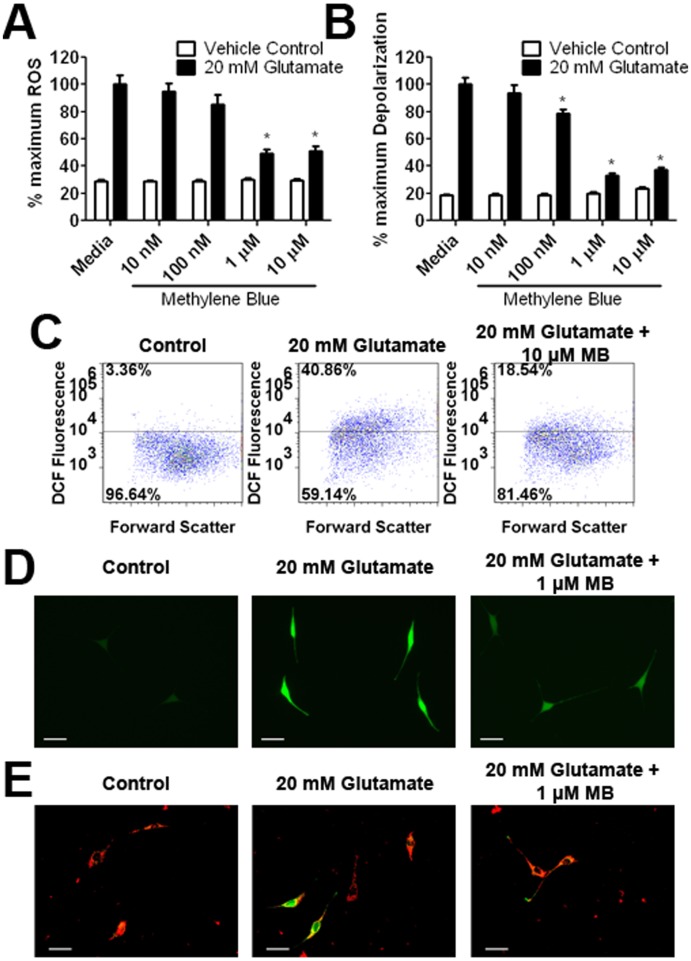
Effect of MB on ROS production and mitochondrial membrane potential depolarization induced by glutamate in HT-22 cells. (A) DCF microplate reader assay depicts that a significant increase of ROS was induced by a 12 hour exposure to 20 mM glutamate, which was dose dependently attenuated MB. (B) TMRE/NAO plate reader assay depicts a significant mitochondria membrane potential depolarization induced by glutamate insult, which was dose dependently attenuated by MB. (C) Representative DCF flow cytometry assay depicts increase of ROS induced by 8 hour exposure of 10 mM glutamate which was attenuated by 10 µM MB. (D) Representative images of DCF fluorescence demonstrated increased cellular ROS after 8 hours exposure to 20 mM glutamate. DCF fluorescence was reduced with co-treatment of 1 µM MB (scale bar 50 µm). (E) Representative images of JC-1 fluorescence indicate mitochondria membrane potential collapse after an 8 hour exposure to 20 mM glutamate, which was attenuated by co-treatment of 1 µM MB (scale bar 50 µm). * p<0.05 compared to 20 mM glutamate media.

### Rotenone Neurotoxicity Assay

HT-22 cells were seeded into 96-well flat-bottomed plates at a density of 3000 cells/well in 100 µl DMEM (high glucose, 1 mM pyruvate, 10% FBS) and allowed to attach overnight. Varying concentrations of MB or its derivatives and 5 µM rotenone (or media for control wells) was then added to each well. Plates were incubated for 24 hours at 37°C with 5% CO_2_. Viability was determined by Calcein AM assay.

**Figure 4:**
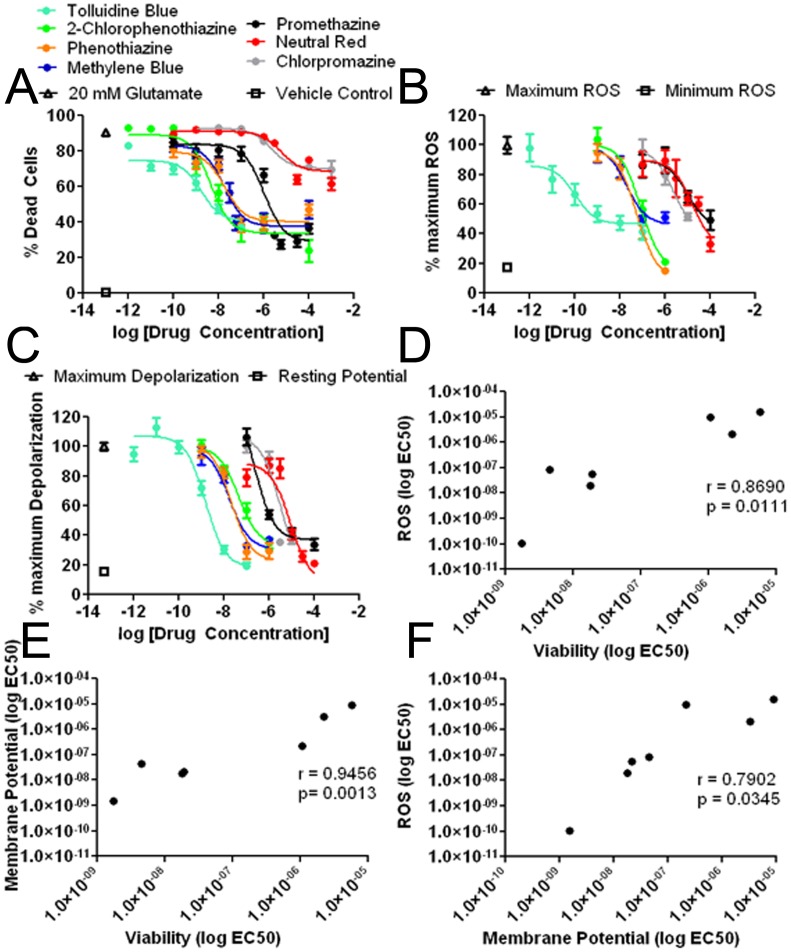
Dose response curves of MB and derivatives in the HT-22 glutamate model. (A) Dose response curves of MB and derivative against glutamate-induced neurotoxicity measured by Calcein AM; (B) Dose response curves of MB and derivatives against glutamate-induced cellular ROS production measured by DCF assay; (C) Dose response curves of MB and derivatives against mitochondria membrane potential depolarization induced by glutamate measured by NAO/TMRE FRET assay; (D) Correlation of cellular ROS production and cell viability, Pearson coefficient  = 0.8690, p  = 0.00111; (E) Correlation of mitochondria membrane potential and cell viability, Pearson coefficient  = 0.9456, p  = 0.0013; (F) Correlation of cellular ROS production and mitochondria membrane potential, Pearson coefficient  = 0.7902, p  = 0.0345.

### Glucose Oxidase Neurotoxicity Assays

HT-22 cells were seeded into 96-well flat-bottomed plates at a density of 3000 cells/well in 100 µl DMEM (high glucose, 1 mM pyruvate, 10% FBS) and allowed to attach overnight. Varying concentrations of MB or its derivatives and 2 U glucose oxidase (or media for control wells) was then added to each well. Plates were incubated for 3 hours at 37°C with 5% CO_2_. Viability was determined by Calcein AM assay.

**Figure 5:**
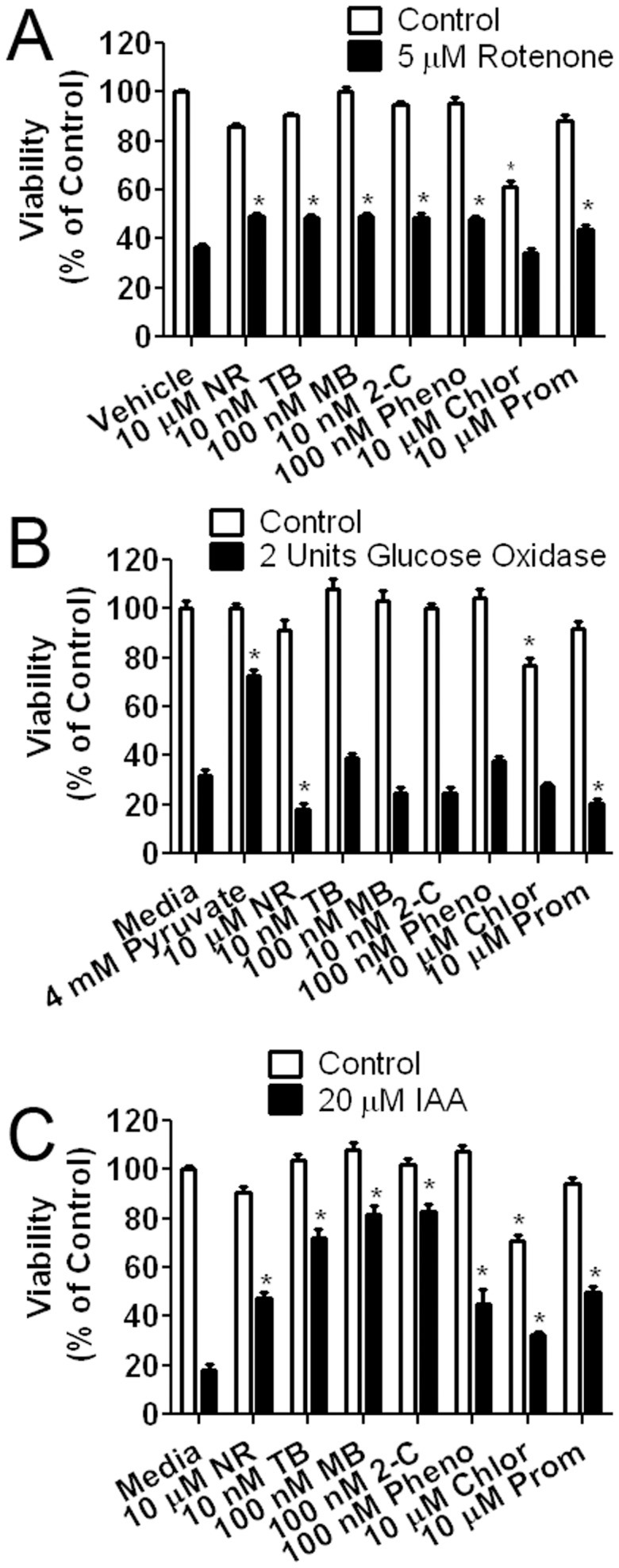
Effect of MB and its derivatives in rotenone, IAA, and glucose oxidase toxicity assays. (A) Effect of MB and derivatives against rotenone neurotoxicity in HT-22 cells. Cells were exposed to 5 µM rotenone for 24 hours in the presence of MB or its derivatives. Protective effect was observed in all compounds except chlorpromazine at the indicated concentrations (NR – Neutral Red; TB – Toluidine Blue O; MB – Methylene Blue; 2-C –2-chlorophenothiazine; Pheno – Phenothiazine; Chlor – Chlorpromazine Prom – Promethazine); # p<0.05 compared to media control. * p<0.05 compared to 5 µM rotenone in media (B) Effect of MB and derivatives against glucose oxidase neurotoxicity in HT-22 cells. Cells were exposed to 2 U glucose oxidase for 3 hours in the presence of MB or derivatives. No protective effect was observed in all compounds tested. Pyruvate (4 mM) was used as a positive control. # p<0.05 compared to media control. * p<0.05 compared to 5 U Glucose Oxidase in media (C) Effect of MB and derivatives against IAA neurotoxicity in HT-22 cells. Cells were exposed to 20 µM IAA for 24 hours in the presence of MB or derivatives. Protective effect was observed in all compounds at the indicated concentration. # p<0.05 compared to media control. * p<0.05 compared to 20 µM IAA in media.

### Iodoacetic Acid (IAA) Neurotoxicity Assays

HT-22 cells were seeded into 96-well flat-bottomed plates at a density of 3000 cells/well in 100 µl DMEM (high glucose, 1 mM pyruvate, 10% FBS) and allowed to attach overnight. Varying concentrations of MB or its derivatives and 20 µM IAA (or media for control wells) was then added to each well. Plates were incubated for 2 hours at 37°C with 5% CO_2_. After 2 hours, all media was removed and replaced with fresh media containing drugs, but not IAA. The plates were incubated an additional 22 hours at 37°C with 5% CO_2_. Viability was determined by Calcein AM assay.

**Figure 6:**
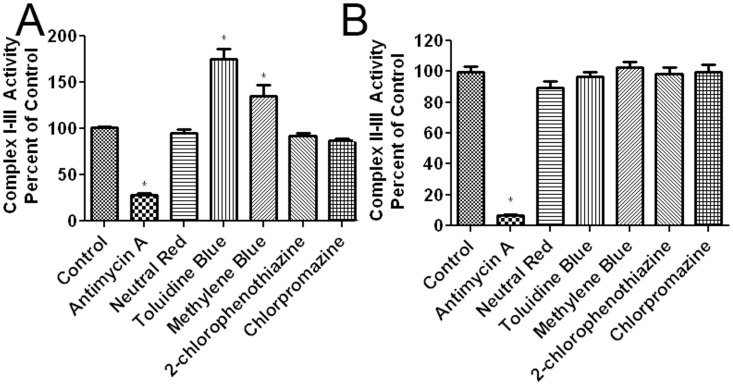
Effect of MB and derivatives on mitochondrial complexes activity. (A) The effects of MB and its derivatives on the rate of cytochrome c reduction were recorded by measuring the change in absorbance of reduced cytochrome c with NADH as an electron donor (complex I-III activity) in isolated mitochondria. All compounds were tested at the concentration of 10 µM. MB and TB significantly increased complex I-III activity (the rate of cytochrome c reduction). Antimycin A (2 µg/ml), a complex III inhibitor, was used as a negative control. (B) Mitochondria complex II-III was measured by the change in absorbance of reduced cytochrome c with succinate as an electron donor in isolated mitochondria. All compounds were tested at the concentration of 10 µM. No effect was observed in all MB related compounds. Antimycin A (2 µg/ml) significantly decreased the rate of cytochrome c reduction. * p<0.05 compared to control.

### Mitochondria Membrane Potential Analysis

Mitochondrial membrane potential was analyzed by FRET using TMRE/NAO as described previously [18]. TMRE quenches the NAO fluorescence under normal mitochondria membrane potential. As the membrane potential collapses, the TMRE fluorescence decreases, which results in an increase in NAO fluorescence. The increased NAO fluorescence is interpreted as a decrease in the mitochondria membrane potential. Cells were incubated with glutamate and MB or related compounds for 12 hours. The media was then removed and the cells were washed once with PBS, then incubated in PBS containing 1 µM NAO and 1 µM TMRE for 30 minutes at 37°C. The NAO/TMRE was removed and cells were incubated for an additional 15 minutes at 37°C in KRH. Cells were washed twice in PBS and NAO fluorescence was measured using a Tecan Infinite F200 plate reader (excitation 485, emission 530). Raw data are represented as RFU. The NAO fluorescence was then standardized based on control and Calcein AM cell viability.

**Figure 7:**
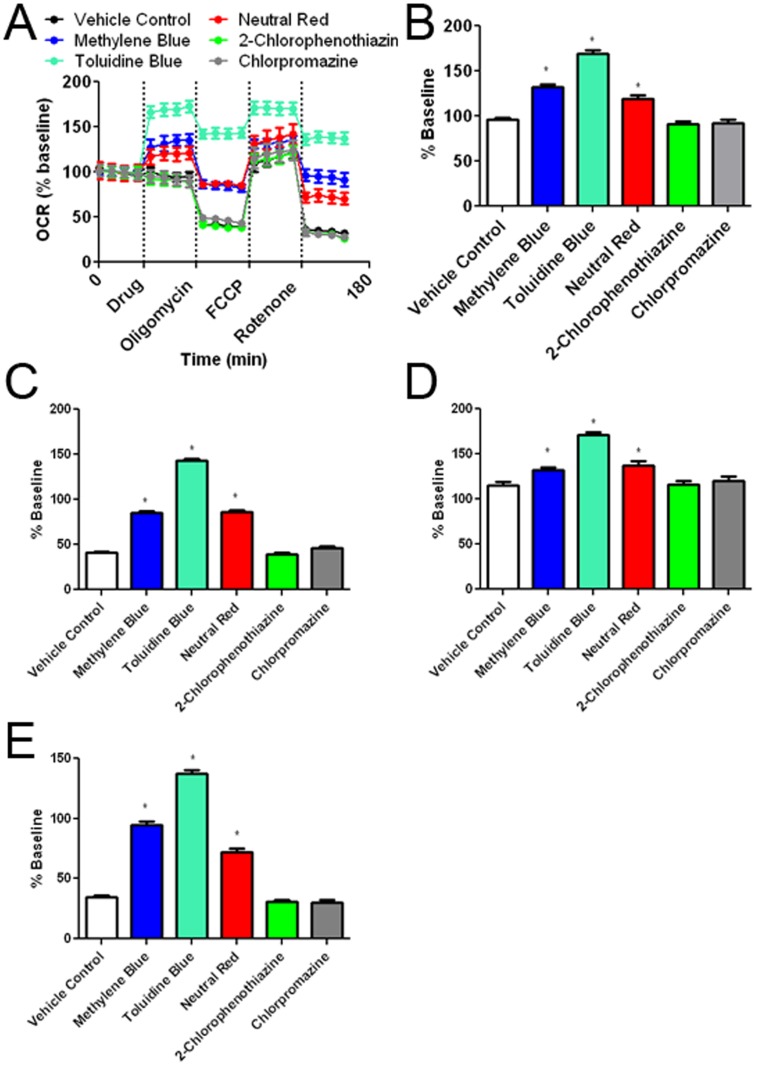
Effects of the MB and its derivatives on cellular oxygen consumption rate (OCR). (A) OCR recording at baseline and subsequent treatment of MB or its derivatives, oligomycin, FCCP, and rotenone. The initial 35 minutes establishes a baseline reading, followed by addition of each drug at a concentration of 10 µM. Three subsequent injections followed consisting of 1 µg/mL oligomycin (complex V inhibitor), 300 nM FCCP (proton gradient uncoupler), and 100 nM rotenone (complex I inhibitor). After each injection, 4 time points were recorded with about 35 minutes between each injection. (B) MB, TB, and NR increased oxygen consumption as compared to vehicle control. 2-Chlorophenothiazine and chlorpromazine had no effect compared to vehicle. (C) Oligomycin decreased cellular oxygen consumption under all experimental conditions. Despite the oligomycin insult, MB, TB and NR significantly increased OCR as compared to vehicle control. (D) Injection of FCCP results in maximum cellular OCR. MB, NR, and TB treated groups have higher maximal respiration than vehicle control. (D) Rotenone inhibits complex I causing a decrease in OCR, which was significantly attenuated by the treatment of MB, NR, and TB. * p<0.05 compared to control group.

Mitochondrial membrane potential was also analyzed by flow cytometry and fluorescent microscopy using JC-1 dye. For fluorescent microscopy, HT-22 cells were plated at a density of 10,000 cells/well in a 6-well plate. Cells were incubated for 8 hours in glutamate and indicated drug. After 6 hours, media was replaced with KRH media containing 5 µg/ml JC-1 dye. Cells were incubated at 37°C for 15 minutes. After which time, they were washed once with KRH and incubated an additional 10 minutes in KRH at 37°C. The media was replaced with fresh KRH buffer and the cells imaged.

**Figure 8:**
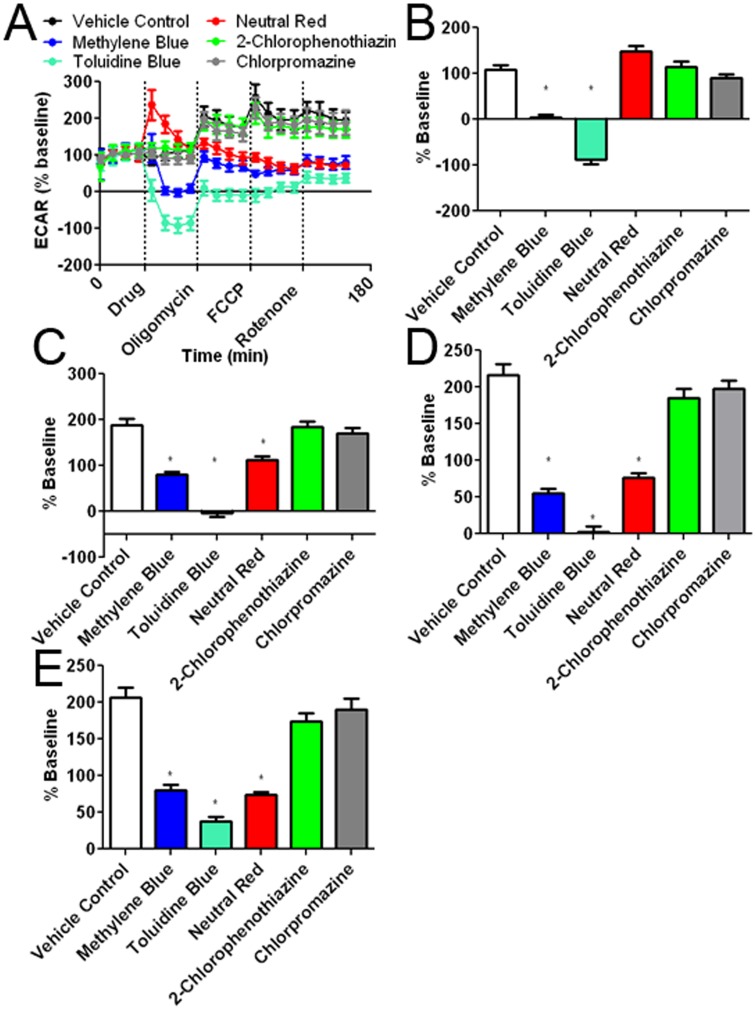
Effects of the MB and its derivatives on extracellular acidification rate (ECAR). (A) ECAR recording at baseline and subsequent addition of MB or its derivatives, oligomycin, FCCP, and rotenone. The initial 35 minutes establishes a baseline reading, followed by addition of drug at a concentration of 10 µM. Three subsequent injections followed consisting of 1 µg/ml oligomycin (complex V inhibitor), 300 nM FCCP (proton gradient uncoupler), and 100 nM rotenone (complex I inhibitor). After each injection, 4 time points were recorded with about 35 minutes between injections. (B) MB and TB decreased ECAR as compared to the vehicle control. 2-Chlorophenothiazine and chlorpromazine had no effect as compared to the control. NR, had no effect to vehicle on average, although a spike in ECAR values was observed after NR injection. (C) Oligomycin increased ECAR under all experimental conditions and was significantly reduced by MB, TB, and NR. (D) Injection of FCCP results in maximum cellular OCR with little effect on ECAR; MB, NR, and TB significantly decreased ECAR as compared to the vehicle control. (D) Rotenone inhibits complex I and decreases OCR without change in ECAR. MB, NR and TB caused ECAR to remain significantly less as compared to the vehicle control. * p<0.05 compared to control group.

**Table 2:** Redox potential of the MB related compounds.

Drug	Redox Potential(s) (E_1/2_ reported in V)	
Neutral Red	0.450	−0.269
Methylene Blue	0.500	−0.108
Toluidine Blue	0.488	−0.012
Phenothiazine	1.342	0.779
2-Chlorophenothiozene	0.942	
Chlorpromazine	1.059	
Promethazine	1.069	

### Reactive Oxygen Species Analysis

Changes in cellular ROS were measured by the ROS reactive fluorescent indicator H_2_DCFDA (Anaspec) using a fluorescent microplate reader, flow cytometry, and fluorescent microscopy. For the microplate experiment, HT-22 cells were plated overnight at a density of 3,000 cells/well in a 96-well plate. Cells were incubated with drug and 20 mM glutamate for 12 hours at 37°C and 5% CO_2_. The media was then removed and the cells were washed once with PBS then incubated in PBS containing 10 µM H_2_DCFDA for 30 minutes at 37°C. The PBS was removed and cells were incubated for an additional 15 minutes at 37°C in KRH. Cells were washed twice in PBS and DCF fluorescence was measured using a Tecan Infinite F200 plate reader (excitation 485, emission 530). Raw data are represented as RFU. The DCF fluorescence was then standardized based on control and Calcein AM cell viability. For fluorescent microscopy, HT-22 cells were plated at a density of 10,000 cells/well in a 6-well plate. Cells were incubated for 8 hours in glutamate and indicated drug. After 8 hours, media was replaced with KRH media containing 10 µM H_2_DCFDA. Cells were incubated at 37°C for 15 minutes, washed once with KRH and incubated an additional 10 minutes in fresh KRH at 37°C. The media was replaced with fresh KRH buffer and the cells imaged. For flow cytometry, HT-22 cells were seeded at a density of 50,000 cells/well in 6-well dishes (Greiner) and attached overnight. Media was removed and replaced with fresh DMEM (high glucose, 1 mM pyruvate, 10% FBS) containing vehicle, 10 µM MB, 20 mM glutamate, or 10 µM MB and20 mM glutamate. Cells were incubated for 8 hours at 37°C and 5% CO_2_. Following the incubation, the media was removed, the cells were washed once with PBS, and incubated in PBS containing 10 µM H_2_DCFDA for 15 minutes at 37°C. The PBS was removed and cells were incubated for an additional 10 minutes at 37°C in PBS. The PBS was replaced with fresh PBS and the DCF fluorescence was determined with a Beckman Coulter FC-500.

**Figure 9:**
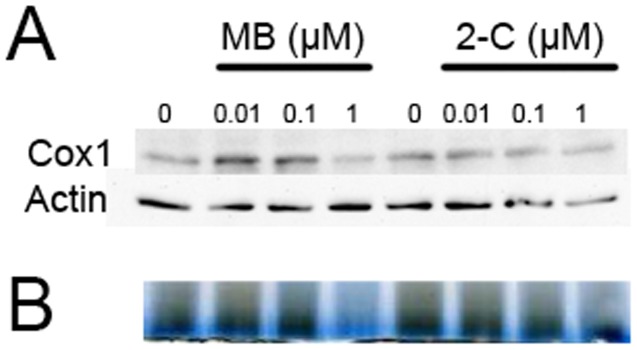
Different action of MB and 2-chlorophenothiazine on mitochondrial complex IV. (A) Western blots depict the expression of complex IV subunit I (Cox1) in HT-22 cells treated with MB or 2-chlorophenothiazine at the indicated concentrations for 3 days. MB, at concentrations of 10 and 100 nM, increased Cox1expression. 2-Chlorophenothiazine had no effect on Cox1 expression at 10 nM, 100 nM, and 1 µM. (B). Blue native indicated an increase in complex IV activity at 100 nM MB and a decrease in complex IV activity at 1 µM MB corresponding to the increased expression of Cox1. 2-Chlorophenothiazine had no effect on complex IV activity at all concentrations tested.

### Glutathione Assay

Glutathione was measured using the Anaspec Total GSH Assay Kit (cat#: 72153). HT-22 cells were seeded into 10 cm plates at a density of 2.5×10^5^ cells/plate. Cells were allowed to attach overnight. Media was removed and replaced with fresh media containing varying concentrations of drug and incubated at 37°C and 5% CO_2_ for 8 hours. Afterwards, the cells were trypsinized for 5 minutes, placed in 1.5 ml Eppendorf tubes, and centrifuged at 1200×g for 5 minutes at 4°C. The cells were centrifuged and washed with PBS twice more, and finally lysed with 100 µl lysis buffer (500 mM Tricine buffer, pH 7.8, 100 mM MgSO_4_, 2 mM EDTA, and 2 mM sodium azide) containing 1% Triton X-100. For protein assays, 40 µl cell lysate was set aside. The remaining cell lysate (60 µl), was combined in a 1∶1 ratio with 2% 5-sufosalicylic acid and centrifuged for 5 minutes at 14,000×g. 10 µl of cell lysate was added in triplicate to a 96-well plate along with a GSH standard curve. Prior to reading the plate, 90 µl reaction buffer containing the following reagents; NADPH, GSH reductase, and diphenyl diselenide was added to each well. Absorbance (415 nm) was measured using a Tecan Infinite F200 plate reader. Protein concentration was measured simultaneously using the Pierce 660 nm Protein Assay (660 nm absorbance).

**Figure 10:**
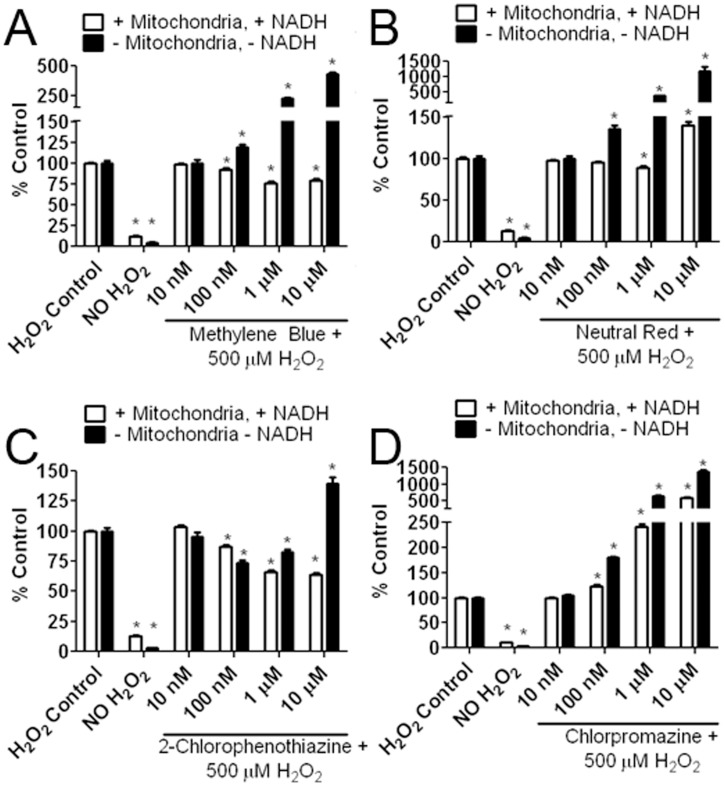
Different action of MB and derivatives as antioxidants. Four compounds were assayed in the presence or absence of mitochondria lysate and 165 µM NADH to determine their effectiveness in mitigating H_2_O_2_ (500 µM) induced DCF oxidation. (A) In the presence of mitochondria lysate and NADH, MB significantly reduced DCF fluorescence at 100 nM, 1 µM and 10 µM. At the same concentrations (100 nM, 1 µM, and 10 µM) in the absence of mitochondria and NADH, MB increased DCF fluorescence. (B) In the presence of mitochondria and NADH, NR decreased DCF fluorescence at a concentration of 1 µM and increased DCF fluorescence at a concentration of 10 µM. NR significantly increased DCF fluorescence at concentrations of 100 nM, 1 µM, and 10 µM in the absence of mitochondria lysate and NADH. (C) 2-Chlorophenothiazine significantly reduced DCF fluorescence at concentrations of 100 and 1 µM in both the presence and absence of mitochondria lysate and NADH. At a concentration of 10 µM, in the presence of mitochondria lysate and NADH, 2-chlorophenothiazine reduced DCF fluorescence; however, in the absence of mitochondria lysate and NADH, 2-chlorophenothiazine increased DCF fluorescence at 10 µM. (D) Chlorpromazine significantly increased DCF fluorescence at concentrations of 100 nM, 1 µM, and 10 µM in both the presence and absence of mitochondria lysate and NADH. * p<0.05 compared to respective H_2_O_2_ control group.

### Mitochondria Isolation and Mitochondrial Complex Activity Assay

Rat hearts were harvested from 3-month old Sprague Dawley rats after euthanasia following the University of North Texas Health Science Center’s IACUC approved protocol; specifically, rats were anesthetized with ketamine/xylazine and death induced by decapitation (guillotine). Rat hearts were flash frozen in liquid nitrogen and stored at −80°C until use. Hearts were thawed on iced and were then homogenized in 10 mM phosphate buffer (pH  = 7.4) containing 300 mM sucrose, and 2 mM EDTA. The resulting homogenate was centrifuged at 800×g and the supernatant collected. The supernatant was then centrifuged at 8,000×g and the resulting pellet containing the mitochondria fraction was re-suspended in 100 mM phosphate buffer (pH  = 7.4). Mitochondria were sonicated 3 times for 30 seconds on low power to break apart mitochondria membranes and expose the individual complexes of the electron transport chain. For complex I/III assay, mitochondria membrane fractions were added to 50 mM phosphate buffer (pH  = 7.4) containing 2 mM MgCl_2_, 2 mM KCN, 80 µM oxidized cytochrome c, and 4 µM NADH. Changes in absorbance at 550 nm were monitored with a spectrophotometer. The addition of 2 µg/ml rotenone was used to inhibit complex I activity. For complex II/III assay, mitochondria membrane fractions were added to 50 mM phosphate buffer (pH  = 7.4) containing 20 mM succinate, 500 µM EDTA, 2 mM KCN, 30 µM oxidized cytochrome c, and 2 µg/ml rotenone. Changes in absorbance at 550 nm were monitored with a spectrophotometer. The addition of 2 µg/ml antimycin was used to inhibit complex III activity.

**Figure 11:**
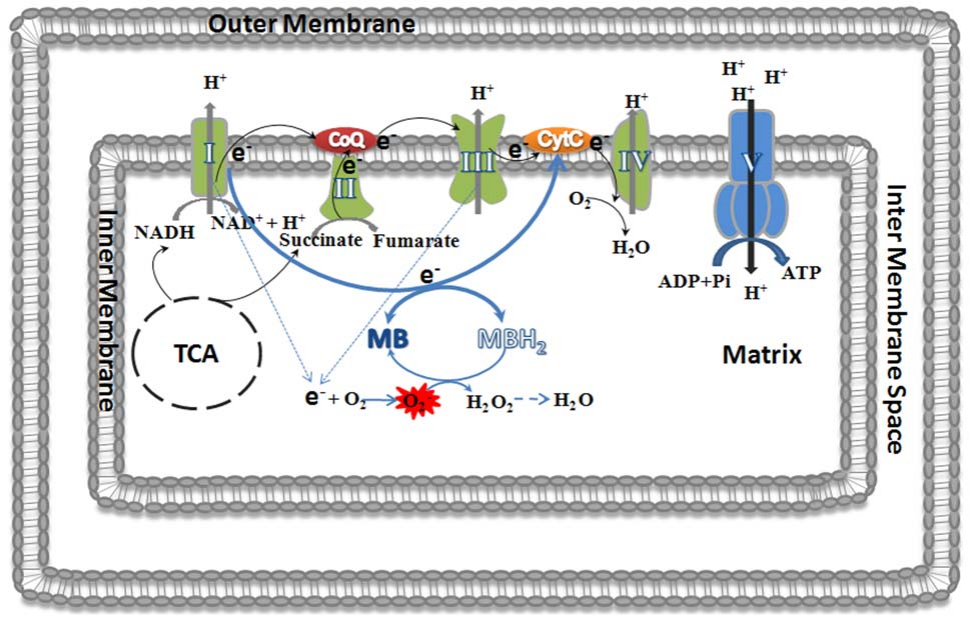
Schematic illustration depicts the novel neuroprotective mechanism of MB. MB receives electrons from NADH through mitochondrial complex I and is reduced to leuco-MB, which can donate the electrons to cytochrome c and recycle to its oxidized form, MB. In addition, the reduced leuco-MB can also function as free radical scavenger and neutralize superoxide generated due to the blockage of complex I and III.

### Cyclic Voltammetry (Redox Potential Analysis)

Cyclic voltammograms were acquired at room temperature using a BASiC3 potentiostat equipped with a 3.0 mm glassy carbon working electrode, a platinum wire auxiliary electrode, and Ag/AgNO_3_ reference electrode. Measurements were performed under a blanket of nitrogen in acetonitrile with [Et4N][BF4] (0.1 M) as supporting electrolyte at a scan rate of 100 mV/s. Analyte concentration was kept constant at 10 mg of sample in 10 ml of solution. Ferrocene was used as an internal standard and reported relative to NHE (Fc/Fc+  = 692 mV vs. NHE).

### Cellular Bioenergetics Assay

HT-22 cells were plated at a density of 5000/well in an XF24 plate. Cells attached overnight and the media was exchanged 1 hour prior to the assay for XF24 media. Rotenone (100 nM), carbonyl cyanide-p- FCCP (300 nM), and oligomycin (1 µg/ml) were diluted into XF24 media and loaded into the accompanying cartridge. Injections of the drugs into the medium occurred at the time points specified. Oxygen consumption was monitored using a Seahorse Bioscience XF24 Extracellular Flux Analyzer.

### Mitochondria Lysate Oxidation Assay

Four compounds (MB, NR, 2-chlorophenothiazine, and chlorpromazine) were assayed in 10 mM phosphate buffer (pH = 7.4) with 500 µM H_2_O_2_, 10 µM DCF and in the presence or absence of 165 µM NADH and mitochondria lysate (19.4 µg/ml). Assay took place in Greiner 96-well black plates for 30 minutes at 37°C, at which time the DCF fluorescence was measured with a Tecan Infinite F200 plate reader (excitation 485, emission 530).

### Western Blot

HT-22 cells were plated at a density of 150000/well in a 6-well plate. Cells attached overnight and either MB or 2-chlorophenothiazine was added to the cells the following day at the indicated concentrations. Cells were grown for 3 days and lysed in radioimmunoprecipitation assay (RIPA) buffer with protease and phosphatase inhibitors. Cell lysate was loaded onto a 10% polyacrylamide gel and transferred onto nitrocellulose. Nitrocellulose was incubated with primary antibody overnight at 4°C at the indicated concentrations (Cox1, Invitrogen, 1∶500; Actin, Santa Cruz Biotechnology, 1∶3000). Secondary antibody linked to horseradish peroxidase (Jackson Immunoresearch) was incubated for 2 hours at room temperature (1∶2000 dilution). Chemiluminescence was detected with a UVP Biospectrum 500.

### Mitochondrial Complex IV Activity Analysis by Blue-native PAGE

Complex IV activity was analyzed by an in-gel method as previously described [19]. Briefly, after separation of complex IV from other mitochondrial complexes by blue native gel electrophoresis, gel strips were incubated in 50 ml potassium phosphate buffer (50 mM, pH 7.0) containing 20 mg of 3,3′-diaminobenzidine tetrachloride (DAB) and 50 mg of cytochrome c. When a clear activity-containing band could be visualized, the staining was stopped by transferring the gel strips to a solution containing 8% acetic acid and 10% method. Gel image was documented by an Epson Perfection 1670 scanner.

### Statistic Analysis

All data were presented as mean +/− S.E.M. The significance of differences among groups with one independent variable was determined by one-way ANOVA with a Tukey’s multiple-comparisons test for planned comparisons between groups when significance was detected. The significance of differences among groups where two independent variables presented were determined by two-way ANOVA with a Bonferronni Post test for planned comparisons between groups when significance was detected. For all tests, p<0.05 was considered significant.

## Results

### Effects of MB and its Derivatives Against Oxidative Insults

The protective action of MB was initially characterized in HT-22 cells using a glutamate neurotoxicity model. In HT-22 cells, glutamate blocks the glutamate/cysteine antiporter with saturating concentrations of extracellular glutamate, resulting in depletion of cellular glutathione [20], [21]. In both Calcein AM and MTT assays, MB provided protection in HT-22 cells with an EC50 of 17.81 nM (Figure 2A, B and C, Table 1) without any effect on the glutathione levels (Figure 2D).

To measure the effect of MB on mitigating ROS generation induced by glutamate insult in HT-22 cells, we employed the fluorescent indicator H_2_DCFDA, which is converted into DCF by ROS. In the plate reader assay, glutamate caused a significant increase in DCF fluorescence, which was attenuated by MB with an EC50 of 20.37 nM (Figure 3A, Table 1). The inhibitory action of MB on ROS production in HT-22 cells was verified using flow cytometry and fluorescent microscopy. MB decreased ROS production measured as total cellular ROS by flow cytometry (Figure 3C). Fluorescent microscopy demonstrated that glutamate exposure increased cellular ROS production which was attenuated by treatment of MB (Figure 3D).

High levels of ROS in the mitochondria affect the mitochondria membrane potential, causing loss of the proton gradient and membrane depolarization. A plate reader assay based on the FRET between NAO and TMRE was run in parallel with a cell viability assay to measure the effect of MB on mitochondria membrane potential depolarization induced by glutamate in HT-22 cells. A 12-hour incubation of HT-22 cells with 20 mM glutamate caused a decrease in mitochondria membrane potential, evidenced by an increase in NAO fluorescence, which was attenuated by the treatment of MB with an EC50 of 17.72 nM (Figure 3B, Table 1). We further verified the protective action of MB on mitochondrial membrane potential using JC-1 with fluorescent microscopy. A decrease in the JC-1 polymer and increase in the JC-1 monomer were observed by microscopy after an 8-hour glutamate exposure, which was greatly attenuated with MB treatment (Figure 3E).

We tested MB’s protective action in the rotenone and IAA neurotoxicity models in HT-22 cells. Rotenone reduces oxidative phosphorylation and generates excess ROS by inhibiting mitochondria complex I (NADH oxidase) [22]. HT-22 cells were incubated for 24 hours in media containing 5 µM rotenone and MB ranging in concentration from 1 nM to 10 µM. MB increased cell viability in rotenone treated cells at concentrations of 10 nM, 100 nM, 1 µM, and 10 µM compared to rotenone control (Figure S1A).

IAA is a toxic derivative of acetic acid that inhibits glyceraldehyde 3-phosphate dehydrogenase and disrupts disulfide bonds causing an increase in ROS and lipid peroxidation [23], [24], [25], [26]. MB protected against IAA induced neurotoxicity at concentrations of 100 nM and 1 µM, but lost its protective effect at 10 µM (Figure S2A).

To determine the effect of MB in mitigating extracellular H_2_O_2_ insult, 2 U of glucose oxidase was added to the media of HT-22 cells. The HT-22 glucose oxidase model is defined by generation of extracellular H_2_O_2_ from glucose, where antioxidants, such as pyruvate, are highly effective [27], [28], [29]. Glucose oxidase induced 80% cell death after 3 hours of exposure which was not attenuated by MB at concentrations between 10 nM and 10 µM (Figure S3). Pyruvate, as a positive antioxidant control, provided protection against 2 U of glucose oxidase at a concentration of 4 mM.

To determine the structure-activity relationships of MB’s neuroprotective action, we selected six commercially available compounds and compared their effects to MB in HT-22 cells. These compounds exhibited a wide range of potencies in HT-22 cells against glutamate insult (Figure 4A). Three of the compounds showed a similar protective profile to that of MB with EC50s of 18.99, 4.57, and 1.75 nM for phenothiazine, 2-chlorophenothiazine, and TB, respectively. The two compounds with side chains on their heterocyclic nitrogen, exhibited much lower potencies as compared to the compounds with free nitrogen groups with EC50s against glutamate neurotoxicity of 1067 and 2192 nM for promethazine and chlorpromazine, respectively. Replacement of the heterocyclic sulfur with nitrogen attenuated the protective action of NR compared to TB against glutamate neurotoxicity (Figure 4A, Table 1).

We determined the structure-activity relationships of MB and its derivatives on ROS production induced by glutamate in HT-22 cells. To measure intracellular ROS, we employed a 96-well assay format and measured DCF fluorescence in parallel with a cell viability assay after a 12-hour incubation of 20 mM glutamate. Glutamate caused a significant increase in ROS in HT-22 cells after 12 hours, which was significantly reduced by phenothiazine and 2-chlorophenothiazine with EC50s of 57.21 and 84.14 nM, respectively (Figure 4B, Table 1). TB, again the most potent of the tested compounds, significantly attenuated ROS production induced by glutamate with an EC50 of 0.11 nM (Figure 4C, Table 1). Consistent with the cell viability study, the two compounds with side chains on their 10 nitrogen, chlorpromazine and promethazine, exhibited significantly lower potencies against ROS generation with EC50s of 2148 and 9687 nM, respectively (Figure 4C, Table 1). Similarly, NR also displayed lower potency against ROS production as compared with TB, MB, phenothiazine, and 2-chlorophenothiazine.

We further determined the structure-activity relationships of MB and its derivatives on glutamate induced mitochondria membrane potential depolarization in HT-22 cells. As indicated by the NAO/TMRE FRET assay, a 12-hour incubation with 20 mM glutamate caused mitochondria membrane potential depolarization which was mitigated by TB, MB, phenothiazine, and 2-chlorophenothiazine with the EC50s of 1.54, 17.72, 21.18, and 45.09 nM respectively. Consistent with the cell viability and ROS production assays, promethazine, chlorpromazine, and NR had much lower potencies as compared to TB, MB, phenothiazine, and 2-chlorophenothiazine (Figure 4C, Table 1).

Our further analysis indicated significant correlation between the EC50s for the effect of MB and its derivatives on ROS production and their neuroprotective action (Figure 4D). Similarly, significant correlation was also found between the action of MB and its derivatives on neuroprotection and mitochondria membrane potential (Figure 4E) and between the EC50 of MB and its derivatives on ROS production and mitochondria membrane potential collapse induced by glutamate insult (Figure 4F).

MB and its derivatives were screened at concentrations between 10 nM to 10 µM in the rotenone, IAA, and glucose oxidase neurotoxicity models in HT-22 cells. For the purpose of conciseness, the concentration displayed for each compound in the rotenone, IAA, and glucose oxidase assays is based on the EC50 value calculated from the glutamate assay. All the tested compounds except for chlorpromazine were protective in the rotenone model of cellular toxicity (Figure 5A). For the glucose oxidase insult, pyruvate provided robust protection as a positive antioxidant as predicted. On the other hand, none of the MB related compounds exhibited any protection (Figure 5B). All of the MB related compounds were efficacious in the IAA neurotoxicity assay (Figure 5C). In addition, MB and 2-chlorophenothiazine had increased efficacy compared to chlorpromazine and NR (Figure S2).

### Effects of MB and Derivatives on Mitochondria

We determined the effect of MB and its derivatives on the activity of mitochondria complexes I-III (NADH oxidase and cytochrome c reductase, respectively) and complexes II-III (succinate dehydrogenase and cytochrome c reductase, respectively). Both assays rely on measuring the rate of cytochrome c reduction in terms of changes in cytochrome c absorbance, which is typically reduced by complex III. In the complex I-III assay, with NADH as the electron donor, MB and TB significantly increased the rate of cytochrome c reduction, while no effect was found in the other tested compounds (Figure 6A). To verify that the negative compounds were incapable of increasing the rate of cytochrome c reduction the concentrations of 1 µM and 100 µM were also tested, yielding negative results (data not shown). Antimycin A, a complex III inhibitor, significantly reduced the rate of cytochrome c reduction. For the complex II-III assay using succinate as the electron donor, no effect was observed in all the tested compounds (Figure 6B). As predicted, antimycin A significantly inhibited complex II-III activity.

We determined the effect of MB and its derivatives on cellular oxygen consumption rate (OCR) and extracellular acidification rate (ECAR) using a Seahorse XF24 Flux Analyzer. OCR and ECAR were measured under five conditions. The first set of measurements established a baseline for 35 minutes followed by injection of media containing MB or each of its derivatives. Upon injection, NR, MB and TB increased OCR, while 2-chlorophenothiazine and chlorpromazine had no effect on the OCR (Figure 7A, C). Cells received sequent treatment of oligomycin (an ATP synthase inhibitor), FCCP (mitochondrial uncoupler), and rotenone (complex I inhibitor). As predicted, oligomycin caused a decrease in OCR, FCCP maximized cellular OCR, and rotenone reduced OCR. At each condition, TB, NR, and MB treatment significantly enhanced OCR compared to control (Figure 7A, D, E). As a control, we tested MB in the absence of cells and determined that MB had no effect on oxygen consumption without the presence of cells (Figure S4C).

Changes in cellular oxygen consumption are often mirrored by opposing changes in lactate production [30]. Changes in lactate production are measured by ECAR. MB and TB had corresponding decreases in ECAR compared to controls (Figure 8A, B, C, D and E). Again, in the absence of cells, MB did not affect ECAR other than an initial spike, which is a slight change of pH caused by MB itself (Figure S4D). NR initially increased ECAR, but ECAR subsequently reduced, reaching a similar level as that of MB (Figure 8A, C, D, and E). To verify that NR was not directly affecting the pH of the media, the experiment was repeated in 10 mM HEPES buffer; however, NR continued to increase ECAR upon initial injection (data not shown).

We determined the redox potential of MB and its derivatives and standardized them to a normal hydrogen electrode (NHE). Our analysis indicated that MB, NR and TB have distinct redox potential from the other derivatives (Table 2). Interestingly, these 3 compounds have similar action on cellular oxygen consumption.

### Action of MB and 2-Chlorophenothiazine on Mitochondria Complex IV

We compared the effect of MB and 2-chlorophenothiazine on the expression of mitochondria complex IV subunit I (Cox1). Previous studies have reported that MB increases the activity of complex IV as well as the expression of subunit II of complex IV, coded by mitochondrial DNA [14], [15], [31]. Our data indicates a clear increase in Cox1 expression upon MB treatment at 10 and 100 nM, but not 1 µM (Figure 9A), a similar result has been reported previously [15]. On the other hand, no effect on Cox 1 expression was observed upon the treatment of 2-chlorophenothiazine. Consistently, MB, but not 2-chlorophenothiazine, treatment increases complex IV activity indicated by the in-gel activity staining (Figure 9B).

### Action of MB and Derivatives as ROS Scavengers

The action of MB and its derivatives as ROS scavengers was determined by a cell-free mitochondria lysate oxidation assay. We combined 500 µM H_2_O_2_ and 10 µM H_2_DCFDA in phosphate buffer with the addition of each MB related compound ranging in concentration between 10 nM and 10 µM. Each compound was tested in both the presence and absence of 165 µM NADH and fractionated heart mitochondria for 30 minutes. NADH served as an electron donor and was necessary for MB to reduce DCF fluorescence. MB reduced DCF fluorescence at concentrations of 100 nM, 1 µM, and 10 µM in the presence of mitochondria and NADH. However, without NADH and mitochondria present, MB increased DCF fluorescence at 100 nM, 1 µM and 10 µM (Figure 10A). NR mildly but significantly decreased DCF fluorescence at 1 µM, while, increasing DCF fluorescence at 10 µM in the presence of mitochondria and NADH. In the absence of mitochondria and NADH, NR treatment increased DCF fluorescence up to 15 fold (Figure 10B). 2-Chlorophenothiazine significantly reduced DCF fluorescence at concentrations of 100 nM, 1 µM, and 10 µM in the presence of mitochondria and NADH. Without mitochondria and NADH, 2-chlorophenothiazine significantly decreased DCF fluorescence at the concentrations of 100 nM and 1 µM, but increased DCF fluorescence at the concentration of 10 µM (Figure 10C). Chlorpromazine significantly increased DCF fluorescence at concentrations of 100 nM, 1 µM, and 10 µM in both the presence and absence of mitochondria and NADH (Figure 10D).

## Discussion

MB has been studied sporadically for over 100 years with its initial biological activity uncovered in the 1890s [32]. Recently, discovery of its cognitive enhancing and neuroprotective effects has reinvigorated research into MB. MB and TB’s oxygen enhancing effects were initially observed in aerobic metabolism [33], [34], [35]. Although the initial results were promising, research into the oxygen enhancing properties of MB did not continue until the 1960s, at which time MB’s actions on the electron transport chain were identified and MB was shown to accept electrons from NADH and transfer them to cytochrome c independent of coenzyme Q10 in isolated live mitochondria [12]. Recently, we have demonstrated MB’s neuroprotective action and its relationship to MBs electron shunt [10]. To elucidate the structural characteristics necessary for MBs mechanisms, we have compared MB to a selected group of MB related compounds.

Our results indicate that the MB related compounds can be divided into four groups based on their structure-activity relationships in neuroprotective and bioenergetics assays. The first group consists of compounds containing only the phenothiazine nucleus (phenothiazine and 2-chlorophenothiazine). These compounds were highly efficacious and potent in the IAA, glutamate, and rotenone neurotoxicity assays, but had no effect on anaerobic glycolysis, cellular oxygen consumption or the complex I-III shunt. The second group of compounds are those with amine side chains attached to the 3, 7 carbons of the phenothiazine nucleus (MB and TB). Both MB and TB had high potencies and efficacies in the neurotoxicity assays, coupled with their ability to enhance cellular oxygen consumption and decrease anaerobic glycolysis. Both compounds were also unique in their ability to act as an intermediate between complex I and cytochrome c. The third group was made up of the compounds with a side chain attached to the 10 nitrogen of phenothiazine (chlorpromazine and promethazine). Promethazine and chlorpromazine were less potent in the glutamate, IAA, and rotenone neurotoxicity assays. In addition, promethazine and chlorpromazine are less efficacious in the IAA assay as compared to the two previous groups with the exposed nitrogen motif in the phenothiazine nucleus (MB and phenothiazine). Besides being less potent, neither promethazine nor chlorpromazine had any effect on cellular oxygen consumption, anaerobic glycolysis, or the complex I-III shunt. The fourth group contained only one compound, NR. NR has a substitution of a nitrogen in place of the 5 sulfur yielding a phenazine nucleus with side chains on the 3, 7 carbons. NR had decreased neuroprotective potency relative to MB in the glutamate, rotenone and IAA assays as well as a decreased efficacy in the IAA assay. However, NR was capable of enhancing cellular oxygen consumption, but did not aid in electron transfer between mitochondria complexes I and III.

The addition of a side chain to the 10 nitrogen caused a significant loss of potency and efficacy as demonstrated by the differences between phenothiazine and chlorpromazine in the glutamate, IAA, and rotenone assays. Chlorpromazine and promethazine have previously been reported to have minor protective actions with micromolar potency, which corresponds to our results from the neurotoxicity assays [36], [37]. In addition, the neuroprotective effects of phenothiazine and chlorpromazine were previously compared in a rotenone neurotoxicity assay highlighting phenothiazine’s robust neuroprotection as compared to chlorpromazine’s lack of efficacy [38]. This was later elaborated on *in vivo* in a C elegans model of Parkinson’s disease, with phenothiazine again being highly efficacious [39], [40], [41].

The position 5 sulfur is as equally important as the availability of the free 10 nitrogen motif evidenced by the differences between TB and NR in the glutamate, IAA, and rotenone assays. The substitution of a nitrogen in place of the sulfur in the heterocyclic nucleus of the molecule (phenothiazine backbone replaced with phenazine backbone) significantly decreased both the potency and efficacy of NR as compared with MB.

MB’s neuroprotective effects have been demonstrated in models of Alzheimer’s disease, Parkinson’s disease, stroke, optic neuropathy, and hypoxia [10], [11], [15], [42], [43], [44]. In addition, phenothiazine has been demonstrated to be protective in models of Parkinson’s disease employing rotenone or MPP^+^ [38], [39]. However, previous studies have not compared the effects of MB and phenothiazine together. Our results indicate that MB and phenothiazine have very similar neuroprotective effects due to the availability of their heterocyclic nitrogen and the presence of the position 5 sulfur. The two structural analogs for phenothiazine and MB, 2-chlorophenothiazine and TB respectively, also exhibit nanomolar neuroprotective effects in our neurotoxicity assays. However, our cellular bioenergetics and mitochondria lysate results indicate an apparent difference between MB and phenothiazine.

The distinct neuroprotective action of MB was suggested by our mitochondrial lysate oxidation assay, where MB requires mitochondria and NADH to reduce oxidative stress. We predict that MB accepts electron(s) from NADH via mitochondria complex I and is reduced to leuco-MB, which can act as a direct free radical scavenger and recycle back to the oxidized form of MB. This unique action of MB makes it a mitochondria specific regenerative anti-oxidant. On the other hand, phenothiazine and 2-chlorophenothiazine can function as direct free radical scavengers independent of the presence of mitochondria and NADH. In addition, the enhancement of complex IV expression and activity associated with MB was not observed with 2-chlorophenothiazine indicating a distinct mechanism between these two compounds. With the addition of a side chain to the 10-nitrogen, chlorpromazine enhanced the oxidative reaction independent of the presence of mitochondria and NADH explaining its low neuroprotective potency.

MB has previously been shown to directly accept electrons from NADH, NADPH, and FADH_2_ [10], [15], [41], [45], [46]. We predicted that MB derivatives derive their protective actions by acting in an electron donor/acceptor capacity between mitochondria complexes I-III similar to MB [10], [12], [15], [47]. Surprisingly, only two compounds, MB and TB, were capable of increasing the rate of cytochrome c reduction in our complex I-III assay. The identified action of MB on complex I-III is consistent with its action on mitochondrial oxidative phosphorylation as we published previously [10]. Similar to MB, we also observed that TB and NR increased cellular oxygen consumption and decreased lactate production although the action and pattern of NR on ECAR was different from that of MB and TB. Interestingly, MB, TB, and NR all have similar negative redox potentials. MB, TB, and other phenazine and phenothiazine derivatives have previously been shown to enhance electron transfer in a microbial fuel cell system [48]. MB, NR, and TB have similar structural characteristics distinct from the other derivatives suggesting that the amine side chains are likely the major factor for the negative redox potential of these compounds, thus, their action on oxygen consumption and lactate production. In addition, since NR, with the substitution of a nitrogen for sulfur in the heterocyclic ring, has a very weak neuroprotective effect and does not function as an alternative electron transfer carrier in mitochondria, we predict that the protective effect of MB is likely related to its action on electron transfer independent of its effect on oxygen consumption and lactate production.

In conclusion, our structure-activity relationship study of MB has demonstrated the distinct anti-oxidant properties of MB. MB acts on superoxide generated due to the blockage of the mitochondria electron transport chain by providing an alternative mitochondrial electron transfer carrier to bypass complexes I-III. In addition, reduced leuco-MB can directly scavenge superoxide and recycle back to the oxidized form MB (Figure 11). As a more than one century old drug, MB has been used clinically for the treatment of multiple diseases with well known pharmacokinetics in humans for both acute intravenous and chronic oral administration [49], [50]. These make MB and some of its derivatives ideal candidates for future investigations for the treatment of neurodegenerative diseases.

## Supporting Information

Figure S1 **Effect of MB and its derivatives on rotenone neurotoxicity in HT-22 cells.** Calcein AM cell viability assay after 24 hour exposure of 5 µM rotenone with co-treatment of (A) MB, (B) 2-chlorophenothiazine, (C) NR, or (D) chlorpromazine. * p<0.05 compared to 5 µM rotenone in media.
(TIF)

Figure S2 **Effect of MB and its derivatives on IAA neurotoxicity in HT-22 cells.** (A) Calcein AM cell viability assay after 24 hour exposure of 20 µM IAA with co-treatment of (A) MB, (B) 2-chlorophenothiazine (C) NR, or (D) chlorpromazine. * p<0.05 compared to 20 µM IAA in media.
(TIF)

Figure S3 **No protective action of MB on direct oxidative insult induced by 3 hours exposure of 2 U glucose oxidase.** MB enhances direct oxidative insult induced cell death at 1 and 10 µM. Pyruvate significantly attenuates the direct oxidative damage acting as an ROS scavenger. * p<0.05 compared to 2 U glucose oxidase in media.
(TIF)

Figure S4 **Effects of phenothiazine and promethazine on OCR and ECAR.** (A) OCR and (B) ECAR recording at baseline and cumulative treatment of each drug (MB, phenothiazine, or promethazine), oligomycin, FCCP, and rotenone. Promethazine and phenothiazine had no effect on OCR and ECAR. (C) OCR and (D) ECAR recordings at baseline and cumulative treatment of MB, oligomycin, FCCP, and rotenone. Wells containing media only were used as blank controls. MB dramatically enhances OCR and inhibits ECAR, but exhibited no effect on OCR and ECAR in blank controls.
(TIF)
