# Supplementary figures and images for: Neuroprotective Actions of Methylene Blue and Its Derivatives
Source: PLoS One. 2012 Oct 31;7(10):e48279. doi: 10.1371/journal.pone.0048279 (PMC3485214; doi:10.1371/journal.pone.0048279)

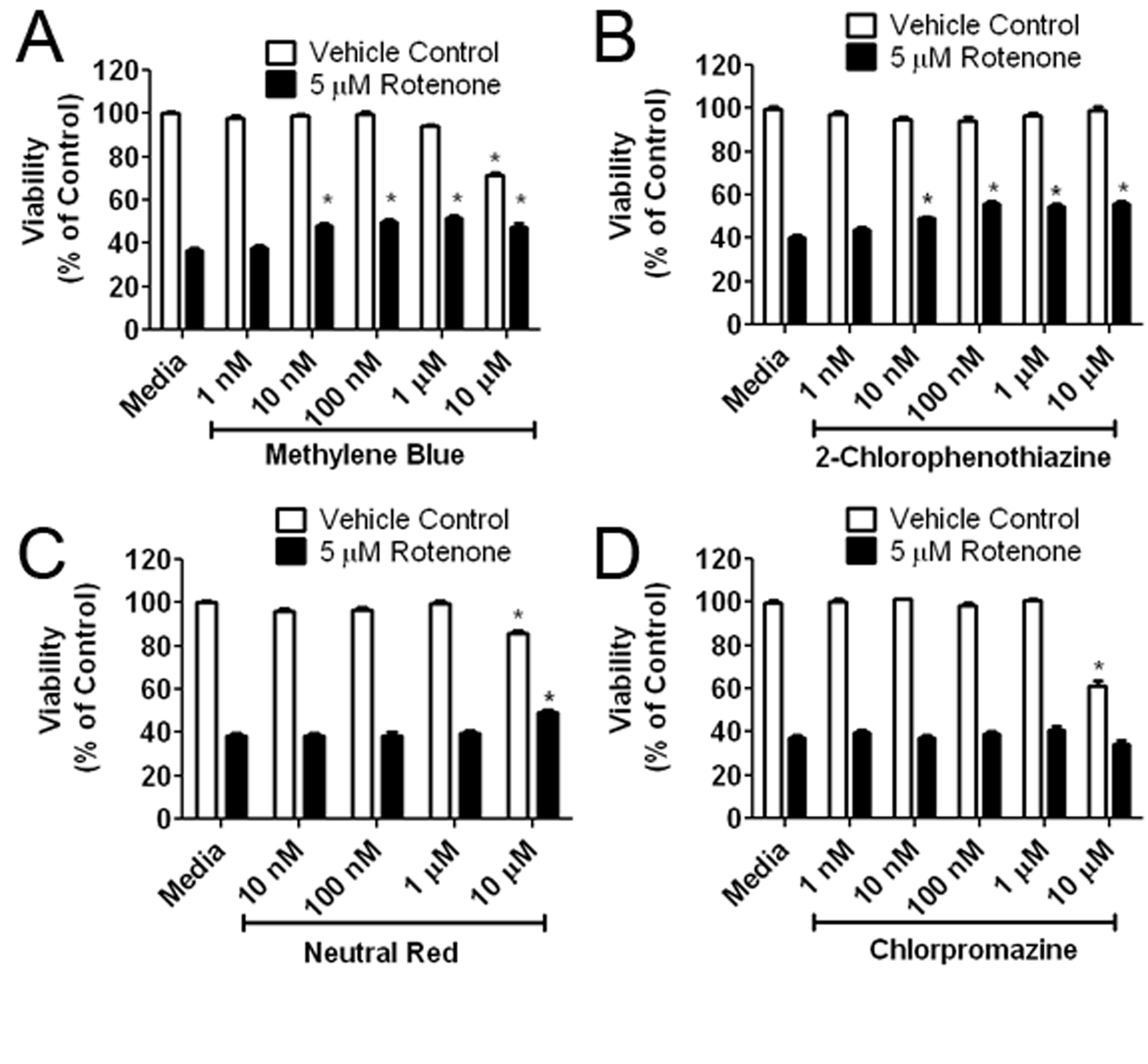

Supplement: Figure S1 — Effect of MB and its derivatives on rotenone neurotoxicity in HT-22 cells. Calcein AM cell viability assay after 24 hour exposure of 5 µM rotenone with co-treatment of (A) MB, (B) 2-chlorophenothiazine, (C) NR, or (D) chlorpromazine. * p<0.05 compared to 5 µM rotenone in media. (TIF) [file pone.0048279.s001.tif]

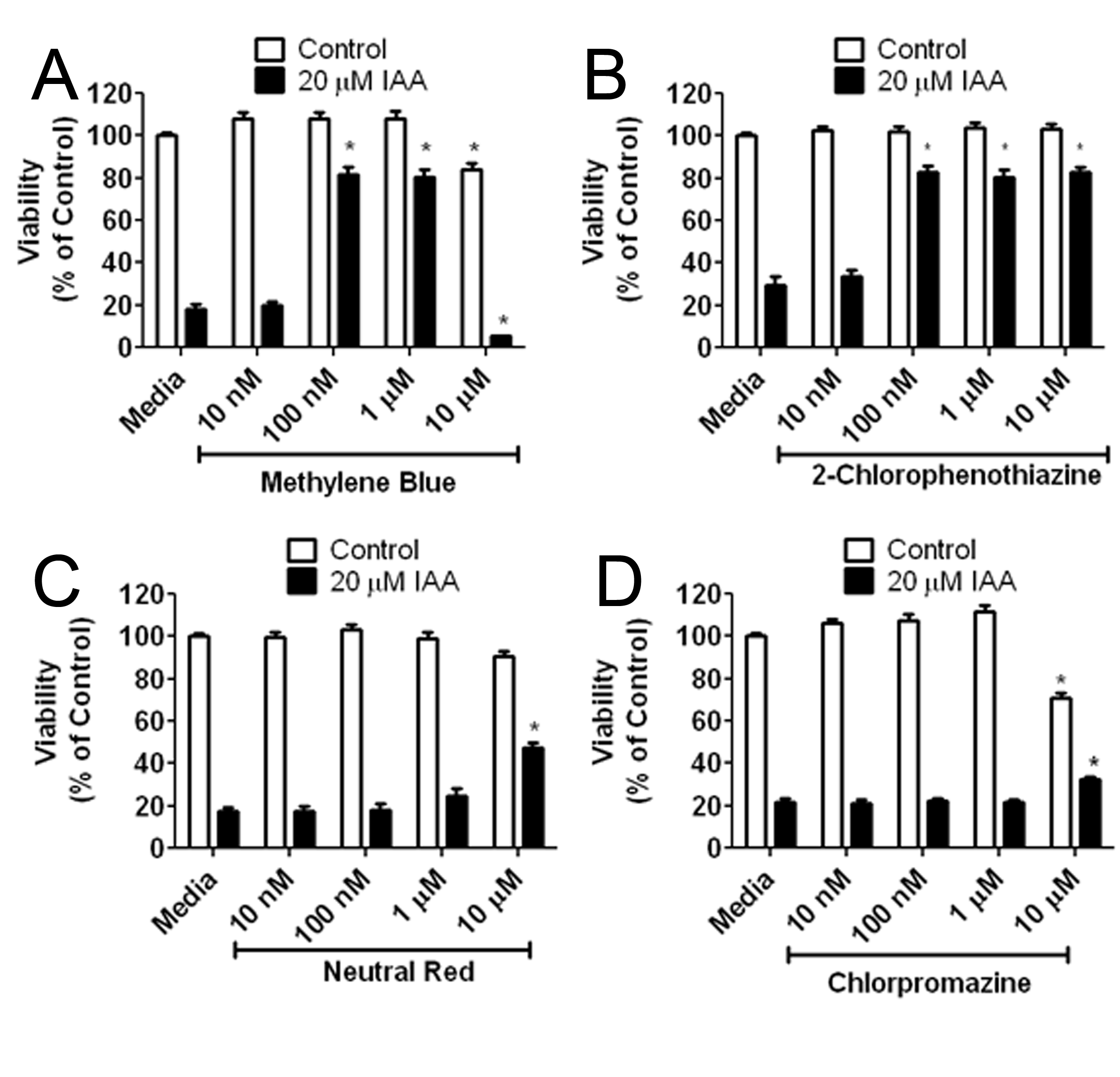

Supplement: Figure S2 — Effect of MB and its derivatives on IAA neurotoxicity in HT-22 cells. (A) Calcein AM cell viability assay after 24 hour exposure of 20 µM IAA with co-treatment of (A) MB, (B) 2-chlorophenothiazine (C) NR, or (D) chlorpromazine. * p<0.05 compared to 20 µM IAA in media. (TIF) [file pone.0048279.s002.tif]

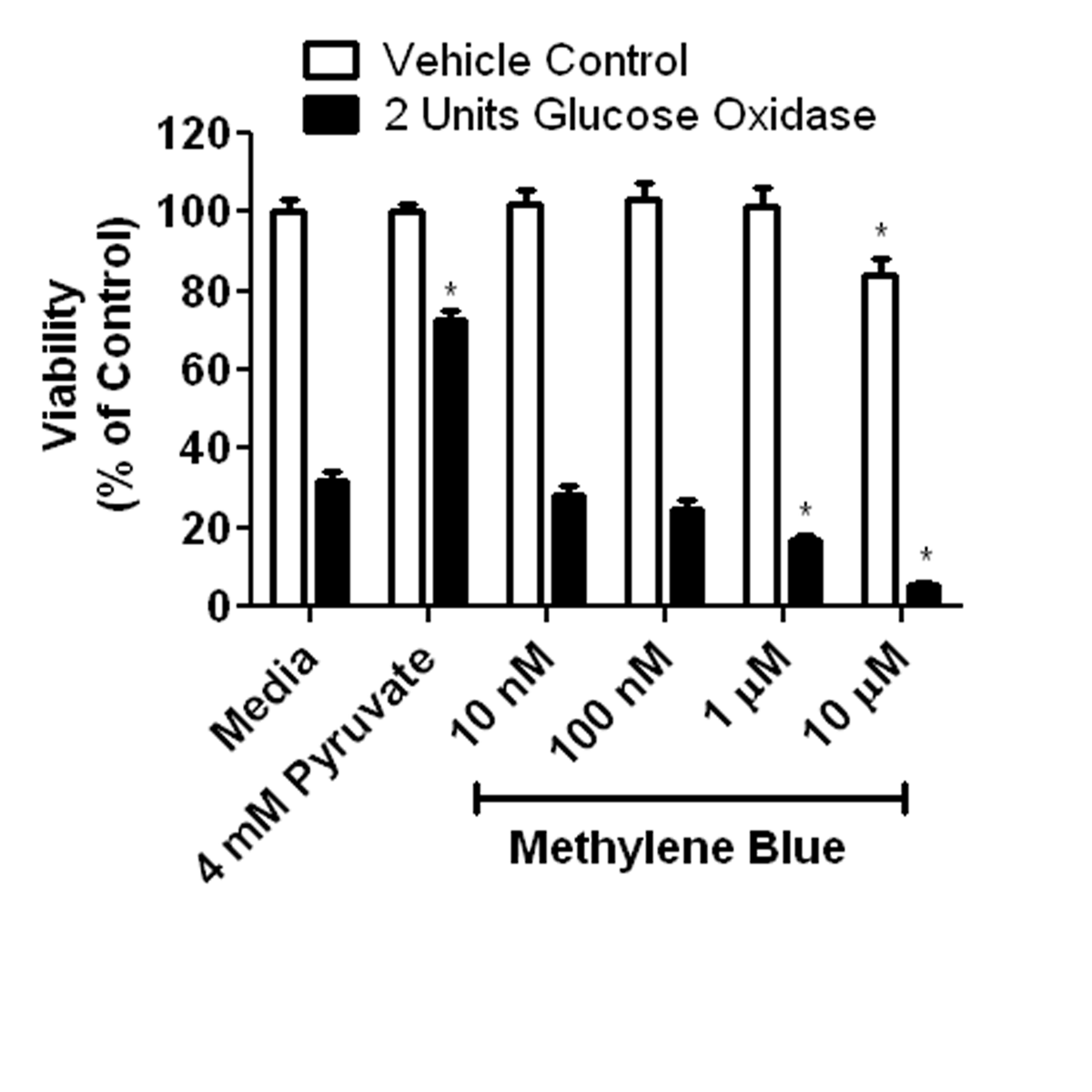

Supplement: Figure S3 — No protective action of MB on direct oxidative insult induced by 3 hours exposure of 2 U glucose oxidase. MB enhances direct oxidative insult induced cell death at 1 and 10 µM. Pyruvate significantly attenuates the direct oxidative damage acting as an ROS scavenger. * p<0.05 compared to 2 U glucose oxidase in media. (TIF) [file pone.0048279.s003.tif]

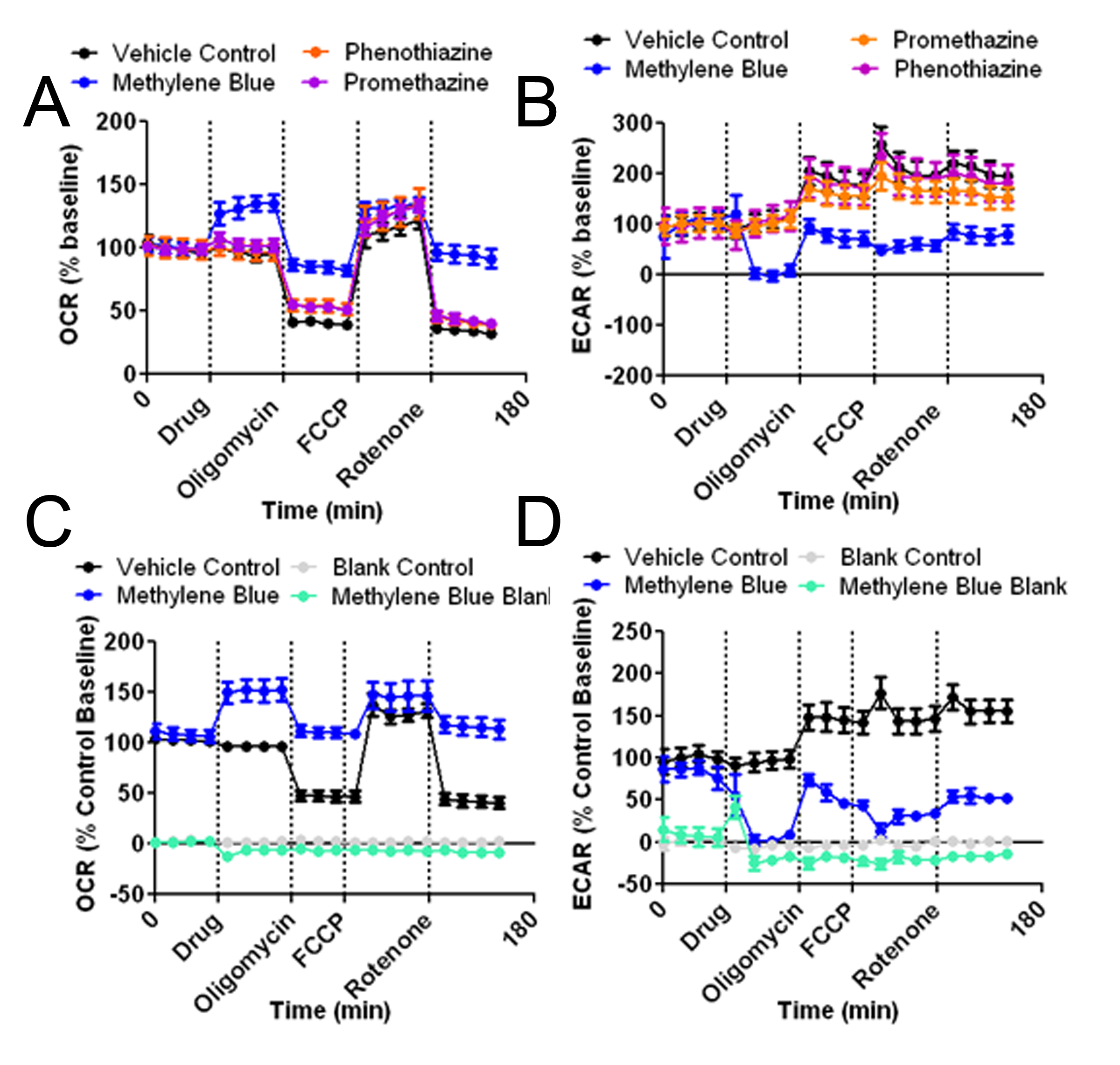

Supplement: Figure S4 — Effects of phenothiazine and promethazine on OCR and ECAR. (A) OCR and (B) ECAR recording at baseline and cumulative treatment of each drug (MB, phenothiazine, or promethazine), oligomycin, FCCP, and rotenone. Promethazine and phenothiazine had no effect on OCR and ECAR. (C) OCR and (D) ECAR recordings at baseline and cumulative treatment of MB, oligomycin, FCCP, and rotenone. Wells containing media only were used as blank controls. MB dramatically enhances OCR and inhibits ECAR, but exhibited no effect on OCR and ECAR in blank controls. (TIF) [file pone.0048279.s004.tif]
